# Restoring a balanced pool of host-derived and microbiota-derived ligands of the aryl hydrocarbon receptor is beneficial after stroke

**DOI:** 10.21203/rs.3.rs-3143015/v1

**Published:** 2023-09-13

**Authors:** Bhanu Priya Ganesh, Pedram Peesh, Maria Pilar Blasco, Ahmad El Hamamy, Romeesa Khan, Gary Guzman, Parisa Honarpisheh, Eric C. Mohan, Grant W. Goodman, Justin N. Nguyen, Anik Banerjee, Kyung Ae Ko, Janelle Korf, Chunfeng Tan, Huihui Fan, Gabriela Colpo, Hilda Ahnstedt, Lucy Couture, Julia Kofler, Jose Moruno-Manchon, Michael Maniskas, Jaroslaw Aronowski, Juneyoung Lee, Jun Li, Robert M. Bryan, Anjali Chauhan, Venugopal Reddy Venna, Louise McCullough

**Affiliations:** McGovern Medical School, The University of Texas Health Science Center at Houston; McGovern Medical School, The University of Texas Health Science Center at Houston; McGovern Medical School, The University of Texas Health Science Center at Houston; McGovern Medical School, The University of Texas Health Science Center at Houston; McGovern Medical School, The University of Texas Health Science Center at Houston; McGovern Medical School, The University of Texas Health Science Center at Houston; McGovern Medical School, The University of Texas Health Science Center at Houston; McGovern Medical School, The University of Texas Health Science Center at Houston; McGovern Medical School, The University of Texas Health Science Center at Houston; McGovern Medical School, The University of Texas Health Science Center at Houston; University of Texas-Houston; McGovern Medical School, The University of Texas Health Science Center at Houston; McGovern Medical School, The University of Texas Health Science Center at Houston; University of Texas Houston; The University of Texas Health Science Center at Houston; The University of Texas McGovern Medical School at Houston, 77030, TX; The University of Texas Health Science Center at Houston; The University of Texas McGovern Medical School at Houston, 77030, TX; University of Pittsburgh; Department of Neurobiology and Anatomy, the University of Texas McGovern Medical School at Houston, 77030, TX; McGovern Medical School, The University of Texas Health Science Center at Houston; University of Texas Health Science Center, McGovern Medical School; The University of Texas Health Science Center at Houston; McGovern Medical School, The University of Texas Health Science Center at Houston; University of Texas-Houston; The University of Texas Health Science Center at Houston; McGovern Medical School/University of Texas Health Science Center at Houston

## Abstract

**Background:**

Stroke is a major cause of morbidity and mortality, and its incidence increases with age. While acute therapies for stroke are currently limited to intravenous thrombolytics and endovascular thrombectomy, recent studies have implicated an important role for the gut microbiome in post-stroke neuroinflammation. After stroke, several immuno-regulatory pathways, including the aryl hydrocarbon receptor (AHR) pathway, become activated. AHR is a master regulatory pathway that mediates neuroinflammation. Among various cell types, microglia (MG), as the resident immune cells of the brain, play a vital role in regulating post-stroke neuroinflammation and antigen presentation. Activation of AHR is dependent on a dynamic balance between host-derived and microbiota-derived ligands. While previous studies have shown that activation of MG AHR by host-derived ligands, such as kynurenine, is detrimental after stroke, the effects of post-stroke changes in microbiota-derived ligands of AHR, such as indoles, is unknown. Our study builds on the concept that differential activation of MG AHR by host-derived versus microbiome-derived metabolites affects outcomes after ischemic stroke. We examined the link between stroke-induced dysbiosis and loss of essential microbiota-derived AHR ligands. We hypothesize that restoring the balance between host-derived (kynurenine) and microbiota-derived (indoles) ligands of AHR is beneficial after stroke, offering a new potential avenue for therapeutic intervention in post-stroke neuroinflammation.

**Method:**

We performed immunohistochemical analysis of brain samples from stroke patients to assess MG AHR expression after stroke. We used metabolomics analysis of plasma samples from stroke and non-stroke control patients with matched comorbidities to determine the levels of indole-based AHR ligands after stroke. We performed transient middle cerebral artery occlusion (MCAO) in aged (18 months) wild-type (WT) and germ-free (GF) mice to investigate the effects of post-stroke treatment with microbiota-derived indoles on outcome. To generate our results, we employed a range of methodologies, including flow cytometry, metabolomics, and 16S microbiome sequencing.

**Results:**

We found that MG AHR expression is increased in human brain after stroke and after *ex vivo* oxygen-glucose deprivation and reperfusion (OGD/R). Microbiota-derived ligands of AHR are decreased in the human plasma at 24 hours after ischemic stroke. Kynurenine and indoles exhibited differential effects on aged WT MG survival after *ex vivo*OGD/R. We found that specific indole-based ligands of AHR (indole-3-propionic acid and indole-3-aldehyde) were absent in GF mice, thus their production depends on the presence of a functional gut microbiota. Additionally, a time-dependent decrease in the concentration of these indole-based AHR ligands occurred in the brain within the first 24 hours after stroke in aged WT mice. Post-stroke treatment of GF mice with a cocktail of microbiota-derived indole-based ligands of AHR regulated MG-mediated neuroinflammation and molecules involved in antigen presentation (increased CD80, MHC-II, and CD11b). Post-stroke treatment of aged WT mice with microbiota-derived indole-based ligands of AHR reduced both infarct volume and neurological deficits at 24 hours.

**Conclusion:**

Our novel findings provide compelling evidence that the restoration of a well-balanced pool of host-derived kynurenine-based and microbiota-derived indole-based ligands of AHR holds considerable therapeutic potential for the treatment of ischemic stroke.

## Introduction

Approximately 800,000 people experience new or recurrent strokes every year^1^. Ischemic stroke, which is caused by a loss of cerebral blood supply due to thrombosis or embolism^2^, is the most common subtype of stroke. Although survival rates after ischemic stroke have improved due to advances in acute management and the use of intravenous thrombolytics such as Alteplase (tissue plasminogen activator) and Tenecteplase, as well as endovascular thrombectomy^3^, these treatments are only available to a small percentage (less than 10%) of stroke patients due to exclusion criteria related to timing, risk of hemorrhage, and the need for specialized resources^4^. Moreover, poor functional outcomes after stroke contribute significantly to a decreased quality of life and the increasing economic burden of stroke^5^. Despite the improved understanding of the immune response after stroke, there are currently no therapeutic options available to regulate neuroinflammation following stroke^6^.

Recent studies have highlighted the crucial role of the gut microbiota and its metabolites in mediating post-stroke immune response through various molecular pathways, including the aryl hydrocarbon receptor (AHR) pathway^7^. AHR is a ligand-activated transcription factor that plays a critical role in immune function and inflammation, and is expressed by a variety of cell types, including T cells, neutrophils, astrocytes, and microglia (MG)^8,9^. Among these cell types, MG, as the resident immune cells of the brain, play a vital role in regulating post-stroke neuroinflammation and antigen presentation.

Stroke is predominantly a disease of aging, and outcome is intricately linked to alterations in MG function with aging^10^. Ongoing investigations into the underlying mechanisms that change with aging have identified gut dysbiosis as a crucial contributor to age-related changes in MG responses. Consequently, both age and stroke-induced dysbiosis can exacerbate the MG response to stroke, highlighting the importance of understanding the mechanisms that link aging, the gut microbiota, and MG-mediated immune processes after stroke^7^. Given that the AHR pathway is a known master regulatory pathway of neuroinflammation and an established molecular sensor of gut microbiota-derived metabolites^9^, exploring the role of MG AHR in the context of age and stroke-induced dysbiosis may help unravel the complex mechanisms that underlie post-stroke inflammation.

The AHR pathway is activated by both exogenous and endogenous ligands, including phytochemicals, metabolic byproducts, host-derived and microbiota-derived ligands^9^. Host-derived AHR ligands are primarily produced by the kynurenine (Kyn) pathway, while microbiota-derived AHR ligands are predominantly produced via the indole pathway^11–14^. Indoles are a class of compounds that are found in plant-based foods (e.g. cruciferous vegetables) and bacterial metabolism of dietary tryptophan (Trp). Indole-mediated activation of AHR creates a dynamic signaling pathway between the gut microbiota and the brain immune compartment in both homeostasis and disease states^15^. Importantly, AHR can mediate both proinflammatory and anti-inflammatory effects in MG depending on the specific ligand profile in the cellular environment^16^. Thus, studying the effects of post-stroke dysbiosis on MG AHR activation is important for understanding the complex mechanisms underlying post-stroke inflammation and identifying new therapeutic targets for stroke. Despite recent advances, the role of microbiota-dependent AHR activation in regulating the post-stroke immune response remains poorly understood.

Plasma concentrations of Kyn increase within hours after a stroke in humans^17^. In mice, host-derived Kyn-dependent activation of AHR after stroke is detrimental, and pharmacological inhibition of AHR can be beneficial, albeit with extensive potential for adverse effects^18^. We hypothesize that stroke-induced dysbiosis leads to a loss of essential microbiota-derived AHR ligands and that restoring the balance between host-derived (Kyn-based) and microbiota-derived (indole-based) ligands of AHR can have therapeutic benefits after stroke. To test our hypothesis, we subjected aged wild-type (WT) and germ-free (GF) mice to transient middle cerebral artery occlusion (MCAO). We utilized metabolomics, flow cytometry, and microbiome sequencing techniques to determine whether post-stroke dysbiosis disrupts the supply of microbiota-derived ligands of AHR in humans. We also sought to determine whether restoring a balanced pool of AHR ligands after stroke could provide therapeutic benefit in aged WT and GF mice. Our findings provide compelling evidence that the restoration of a well-balanced pool of both host-derived and microbiota-derived AHR ligands holds considerable therapeutic potential for the acute treatment of ischemic stroke.

## Methods

### Human case selection and immunohistochemistry (IHC):

Postmortem human brain tissue was obtained from the Neuropathology Core of the Alzheimer’s Disease Research Center (ADRC) at the University of Pittsburgh. The subjects’ information is listed in (**Supp Table S1**). IHC for formalin fixed paraffin embedded human brain sections were performed as described previously^19,20^. Briefly, after deparaffinization, the sections were subjected to heat-induced antigen retrieval process (Citra buffer, pH6.0, 99°C 20min), then blocked in blocking buffer (5% donkey normal serum, 1% BSA with 0.3% Triton X-100 in PBS) for 1 hour at room temperature. The sections were incubated in primary antibodies, Anti-AHR (1:500, NSJ Bioreagents) and anti-Iba1 (1:50, Fujifilm), and then detected by secondary antibodies, Alexa Fluor 488-conjugated Donkey anti-Rabbit and Alexa Fluor 647-conjugated Donkey anti-mouse (1:200, Jackson ImmunoResearch).

### Imaging and quantification of MG AHR expression in human brain samples

Imaging was performed with Leica THUNDER Imager DMi8, using a 40x lens. Five images from both infarct and peri-infarct areas in stroke cases and five images from the normal cortical region in control cases were taken. The infarct and peri-infarct regions were confirmed using hematoxylin and eosin (H&E) staining by a neuropathologist. Images were analyzed using the ImageJ software by an investigator blinded to case conditions. All images were corrected for background noise and consistent fluorescence intensity. Quantities of AHR and Iba-1-positive cells were obtained using individual regions-of-interest (ROIs) within green or red-specific channels, respectively. The quantities of AHR/Iba-1 double-positive cells were measured using overlay images consisting of both channels, and counts were based on reselection of double-positive ROIs. Data are expressed as mean values ± SEM.

### Human MG cell line

HMC3 cells were maintained with EMEM supplemented with 10% FBS and 1% penicillin-streptomycin in a humidified incubator at 37°C and 5% CO2.

### Oxygen-glucose deprivation and reperfusion (OGD/R)

For oxygen and glucose deprivation studies of human MG, cells were washed with PBS and incubated with complete media (normoxia condition) or subjected to oxygen and glucose deprivation (OGD/R; 117 mM NaCl, 5.3 mM KCl, 1.8 mM CaCl2, 0.8 mM MgSO4, 26 mM NaHCO3, and 1.17 mM NaH2PO4, pH 7.4) in a hypoxia incubator chamber for 2 hours at 37°C. Cells were then washed with supplemented EMEM and incubated with the same media for 4 hours^21^. Then, cells were collected in Qizol reagent for further analysis by qPCR^22^. Sorted MG from aged male WT mice were seeded onto 6-well culture plates at a density of 10,000 sorted cells/well. OGD/R was performed as previously reported^23^. Briefly the culture medium was replaced with glucose-free DMEM-A1443001 (Thermo Fisher Scientific), and then the plates were put in a sealed chamber, followed by expiring oxygen for 10 minutes via flowing in 95% N2 and 5% CO2 mixture (Airgas-Southwest Inc.) persistently at a low flow. The chamber was transferred into a 37°C incubator after clamping the inlet and outlet, for 4 hours to mimic OGD/R. In the course of OGD/R, O2 levels dropped to < 2% after 2 hours, and < 1% at 4 hours, as shown by a change in color of BD-271051 anaerobic indicator strips from blue (aerobic) to white (anaerobic) coloration. The medium was changed to normal feeding DMEM-31053036 (ThermoFisher Scientific) and cells restored to a normoxic atmosphere by incubation at 95% oxygen, 5% CO2 and at 37°C as reperfusion for 2 hours.

### Consideration of sex as a biological variable

It has been reported that the interactions between AHR and estrogen receptor can occur independently of estrogen^24^. To avoid this confound, we limited the murine studies to aged male mice. Human samples included both males and females; all human female samples included in this study were from post-menopausal individuals.

### Wild-type (WT) mice

C57BL/6 male mice were obtained from the National Institute on Aging (NIA), Charles Rivers, or Jackson laboratories. Young (3 months) and aged (18 months) were used, respectively. All mice were received at least 8 weeks prior to the experimental date and then aged in house to the appropriate study time points such that microbiome composition was stabilized.

All animals except germ-free (GF) mice were group-housed in Tecniplast individually ventilated cage (IVC) racks, were fed a commercially available irradiated, balanced mouse diet (no. 5058, LabDiet, St Louis, MO) and were provided corncob bedding. Rooms were maintained at 70–73°F and under a 12:12-h light:dark cycle. All animals were maintained specific pathogen free. Animal procedures were performed at an AAALAC accredited facility and were approved by the Animal Welfare Committee at the University of Texas Health Science Center at Houston, TX, USA.

### Germ-free (GF) mice

This study was performed under the guidelines of the National Institute of Health and all experiments were approved by the Institutional Animal Care and Use Committees at the University of Texas Health Science Center at Houston (UTHSCH) and the Baylor College of Medicine. C57BL/6-GF male mice (3 months) were obtained from the Baylor College of Medicine Gnotobiotic Rodent Facility and were shipped in autoclaved shipping crates to UTHSCH. The crates were assembled, bedded with Alpha-Dri bedding and autoclaved with the lid closed. The mice were removed from the isolator in the gnotobiotic facility using sterile transfer bags, were housed in the shipping crates using a biosafety cabinet, and were immediately shipped. The two medical schools are only a few hundred yards apart precluding lengthy and stressful travel. Upon arrival at UTHSCH, fecal samples were collected to confirm the absence of bacteria in the gut. To assess the “germ-free” status of the mice upon arrival, an internal standard was serially diluted and the copy number of the 16S rRNA gene in feces from each transfer crate was analyzed using qPCR. 16S rRNA gene was not detected in feces from any of the transfer crates confirming that the mice were GF. Once the aseptic crates were opened at the UTHSCH, the mice were transferred to autoclaved cages, provided with autoclave chow and water, and maintained in a conventional animal holding room for surgery on the same day. All GF mice were euthanized at 24 hours after surgery.

### Mouse Middle cerebral artery occlusion (MCAO)

Transient focal ischemia was induced under isoflurane anesthesia in young, aged, or GF mice for 60 minutes by occlusion of the right middle cerebral artery^25^. Body temperature was maintained at 37.0 ± 1.0°C throughout the surgery by an automated temperature control feedback system (TC1000, mouse, CWE Inc., USA). A midline ventral neck incision was made, and unilateral MCAO was performed by inserting a Doccol monofilament (Doccol Corp, Redlands, CA, USA) into the right internal carotid artery. One hour after ischemia, animals were re-anesthetized, and reperfusion was established by withdrawal of the monofilament. Animals were then placed in a recovery cage and were euthanized 24 hours after reperfusion. Sham controls were subjected to same procedure except the suture was not introduced into the middle cerebral artery. Animals were randomly assigned into the stroke and sham surgery groups and single housed in their recovery cages for the first two hours after surgery and then returned to group housing. All analyses were performed by investigators blinded to surgical conditions. Sham and stroke mice were housed separately to minimize effects of microbiota contamination between experimental groups. A total of 5 mice were excluded from the study analysis, because of either death during MCAO surgery (n = 2), subarachnoid hemorrhage (n = 1), or no significant intra-ischemic neurological deficits (NDS = 0) after stroke (n = 2).

#### Mouse Neurological deficit score (NDS):

NDS was assessed immediately and 24 hours post-stroke. The Bederson-score system [0 (normal) to 4 (the most severe)] was employed ^26^. Briefly, the scores were assigned according to the following criteria: 0, no deficit; 1, forelimb weakness and torso turning to the ipsilateral side when animals were held by the tail; 2, circling to the affected side; 3, inability to bear weight on the affected side; and 4, no spontaneous locomotor activity or spontaneous barrel rolling. Animals NDS scores of 0 or 4 were after reperfusion were excluded from the study.

### Mouse Infarct Volume Assessment

Mice were euthanized at 24 hours post-MCAO for infarct volume analysis as previously described^27^ with modifications. Briefly, mice were transcardially perfused with ice-cold PBS. Brains were removed to measure weights and volume (by submerging into a PBS containing graduated cylinder for precise volumetric measurements). Brains were sectioned into 5 slices of 1.5 mm thickness using a brain matrix, after removing the olfactory bulb and cerebellum. Slices were immersed in 1.5% 2,3,5-triphenyl-tetrazolium (TTC) in PBS for 10 minutes at 37° C, then fixed in 4% PFA in PBS. Slices were photographed, and the infarct volumes (corrected for edema) were analyzed using Sigma Scan Pro 5 (SPSS Inc.).

### RT-qPCR

To quantify relative mRNA expression levels of *Ahr* and *Cyp1b1* mRNA was extracted from HMC3 cultured cells using the miRNeasy^®^ mini kit (QIAGEN). 2 micrograms of mRNA was reverse-transcribed to single stranded cDNA using the RevertAid H minus First Strand cDNA Synthesis Kit (Thermo Fisher, USA). Reverse transcriptase real-time (RT) PCR was performed using the Quant Studio 3 Real-Time PCR system (Applied Biosystems, USA). The RT-PCR reaction mix (adjusted with H2O to a total volume of 20 μl) contained 1 μl template DNA, 10 μl TaqMan Fast advanced master mix (Thermo Fischer, USA), and 1 μl of the respective primer probes (*Ahr* (Hs00169233_m1), Cyp1b1 (Hs02382916_s1), Gapdh (Hs02758991_g1)). Relative mRNA target gene expression levels (Ratio = [(Etarget) ^dCPtarget (control−sample)^] / [(Eref.) ^dCPref. (control−sample)^]) were normalized to the house keeping gene glyceraldehyde 3-phosphate dehydrogenase (GAPDH) and used as a reference^28,29^.

#### Treatment with indoles cocktail in mice:

IPA and IAld (indole-3-propionic acid and indole-3-aldehyde also known as indole-3-carboxyaldehyde, Sigma Aldrich, Cat #: 220027 and 129445) were dissolved in warmed (37°C) corn oil with less than 1% DMSO to the final concentration of 0.1nmol/g of body weight (approximated based on the tissue metabolomics data obtained from WT mice) for oral gavage, administered as a single dose 3 hours post-reperfusion. The total volume of dissolved cocktail administered orally was 200ul per animal. The vehicle solution was identical in both corn oil and DMSO content but without indole-based molecules.

## Flow cytometry

### Blood (mouse)

Blood was drawn by cardiac puncture with heparinized needles. Red blood cell lysis was achieved by two consecutive 10-min incubations with tris–ammonium chloride (Stem Cell Technologies)^30,31^.

### Blood (mouse)

Mice were transcardially perfused with 60 ml cold, sterile PBS prior to aseptic removal of spleen, and brain tissues. Brain tissue was placed in complete Roswell Park Memorial Institute medium 1640 (Lonza) and mechanically and enzymatically digested in Collagenase/Dispase (1 mg/mL) and DNase (10 mg/mL; Roche Diagnostics) for 45 minutes at 37°C. The cell suspension was filtered through a 70 μm filter. Leukocytes were harvested from the interphase of a 70%-to-30% Percoll gradient^10,32^.

### Skull bone marrow (mouse)

For the isolation of skull bone marrow, meninges were gently peeled from skull cup. The skull was then cut into small pieces using sterile scissors and mechanically dissociated in PBS buffer, followed by a filtration step through a 70-μm cell strainer. After centrifugation at 500g for 5 min, red blood cells were removed by adding 1 ml of ammonium chloride solution (Stem Cell Technologies, Cat #07850) lysis buffer for 2 min at room temperature, centrifugation at 500g for 5 min, and the cell pellet was resuspended in PBS buffer until use.

#### Spleen (mouse):

Spleen (whole) tissue was removed after PBS perfusion and passed through a 70 μm strainer and ammonium chloride solution (Stem Cell Technologies, Cat #07850) in 9:1 ratio was used once for red blood cell lysis.

#### Surface and intracellular staining:

Cells were washed and blocked with mouse FcR Block (BioLegend) before staining with antibodies pre-conjugated with fluorophores (BioLegend): CD45-eF450 (eBioscience, Cat#: 48–0451-82, Lot: 2005853), CD11b-APC (BioLegend, Cat#: 101212, Lot: B279418), Ly6CPerCP-Cy5.5 (BioLegend, Cat#: 128011, Lot: 292026), Tmem119-PE-Cy7 (eBioscience, Cat#: 25–6119-82, Lot: 2210260), P2RY12-PE (BioLegend, Cat#: 848003, B298459), and MHCII-APC-Fire750 (BioLegend, Cat#: 107652, Lot: B301025) pre-conjugated antibodies and Zombie Aqua (BioLegend, Cat#: 423102, Lot: B300004). For intracellular staining, cells were fixed and permeabilized (Biolegend, Cyto-Fast Fix/Perm kit, Cat#: 750000133) following manufacturer’s protocol. Pre-conjugated AHR-BV421 antibody (BD Horizon, Cat#: 565791) was used for intracellular staining. Data were acquired on a Cytoflex-S (Beckman Coulter) cytometer and analyzed using FlowJo (Treestar Inc.). No less than 100,000 events were recorded for each sample/tissue. Cell type-matched fluorescence minus one (FMO) and unstained controls were used to aid in gating strategy. UMAP plots were generated in FlowJo using DownSample v3 plug-in (3,000 Live CD45^+^ cells per sample) followed by UMAP v3.1 algorithm on all uncompensated parameters (except viability) using Euclidean distances, 15 nearest neighbors, minimum distance of 0.5, and 2 components. Following UMAP analysis, Phenograph v3.0 plug-in was used with K value of 174 (recommended by FlowJo) to generate 20 clusters of immune populations. Surface expression heatmaps for CD80, MHC-II, CD11b, P2RY12, and Tmem119 for all clusters are provided in supplementary data (**Supp** Fig. 4). Identification markers for each cluster are also included as supplementary data (**Supp** Fig. 5).

### Cell sorting

Single cell suspension and surface staining were performed as described above. After viability and singlet selections, MG gated as Live Tmem119^+^ (verified to be CD45^int^CD11b^+^) were sorted under an aseptic hood from the single cell suspension prepared from naïve aged male brains (full brains, n = 8) using BD FACSMelody. Sorting was performed from tube directly into 96-well plate used for *ex vivo* experiments to preserve cell counts. Cells were then washed with PBS, stained for surface markers and viability after OGD/R and then analyzed by flow cytometry.

#### Mouse fecal collection and 16S rRNA sequencing:

Microbiota in fecal samples were collected from mice at the same location and time of the day for all groups then stored in sterile tubes at − 80°C until analyzed, as described previously^26^. Bacteria taxa in each fecal sample were analyzed by amplifying the V4 to V5 hypervariable regions of the 16S ribosomal RNA (rRNA) gene using high-throughput sequence analysis performed by Alkek Center for Metagenomics and Microbiome Research (Baylor College of Medicine, Houston, TX). Work flow specifications included: Nucleic Acid Extraction Protocol: MoBIO PowerSoil v3.4, Amplification Protocols: Illumina 16Sv4 v1.2, Sequencing Protocol: Illumina MisSeq v2 2×250 v1.8 ; Illumina, San Diego, CA)^33^, and Analytics Pipeline: CMMR 16Sv4 v1.0. Quality filtered 16S rRNA sequences were clustered into operational taxonomic units (OTUs), with 97% similarity, by closed reference OTU-picking using the UCLUST algorithm and GreenGenes reference database (v13.5) as implemented in Quantitative Insights Into Microbial Ecology (QIIME versions 1.6 and 1.7)^34–36^. Sequences were checked for chimeras using ChimeraSlayer with standard options as implemented in QIIME. Sequences not clustered were identified using the Ribosomal Database Project to the lowest possible taxonomic level^37^.

#### Human and mouse metabolomics analysis (LC-HRMS):

Human plasma samples from stroke and control patients were used. Demographic characteristics of the human plasma samples are included in Supp Table S2. To determine the relative concentration of Trp metabolites in brain tissue and plasma samples, extracts were prepared and analyzed by liquid chromatography coupled with high-resolution mass spectrometry (LC-HRMS). For tissue samples, 100mg of tissue was pulverized on liquid nitrogen, then homogenized with Precellys Tissue Homogenizer. For plasma samples, 100uL of plasma were aliquoted and metabolites were extracted using 0.5 mL ice-cold 50/50 (v/v) methanol/acetonitrile followed by 0.5mL 0.1% formic acid in 50/50 (v/v) Acetonitrile/Water. Extracts were centrifuged at 17,000 g for 5 min at 4°C, and supernatants were transferred to clean tubes, followed by evaporation to dryness under nitrogen. Samples were then reconstituted in 50/50 (v/v) methanol/water, then 10 μL was injected into a Thermo Vanquish liquid chromatography (LC) system containing a Waters XSELECT HSS T3 2.1× 150 mm column with 2.5 μm particle size. Mobile phase A (MPA) was 0.1% formic acid in water. Mobile phase B (MPB) was 100% methanol. The flow rate was 200 μL/min (at 35°C), and the gradient conditions were: initial 5% MPB, increased to 95% MPB at 15 min, held at 95% MPB for 5 min, returned to initial conditions and equilibrated for 5 min. The total run time was 25 min. Data were acquired using a Thermo Orbitrap Fusion Tribrid mass spectrometer under ESI positive and negative ionization modes at a resolution of 240,000 with full scan mode. Raw data files were imported into Thermo Trace Finder software for final analysis. The relative concentration of each compound was normalized by weight for tissue samples and volume for plasma samples.

Signals for indole-based metabolites, Indole-3-propionate and Indole-3-carboxylaldehyde (ChemID 100001083 and 100002185), were extracted, merged, batch-corrected, log-normalized and scaled from two cohorts of stroke patients (n = 60) and healthy controls (n = 64) using R package metabolomicsR^38^. Violin-box plots were utilized to visualize the difference of the above two metabolites between stroke patients and non-stroke age-matched controls. Among stroke patients, linear regression and confidence intervals were constructed using R package ggplot2 between metabolite signals and the NIH stroke scale. R software (version 4.2.2 and higher) was used for metabolic analysis.

### Analysis and statistical methods

Statistical analysis was performed using two-way ANOVA with sex, age, and their interaction as the factors. Main effects were tested, followed by post hoc analysis with all related *p* values adjusted by Sidak’s methods for multiple comparisons. Statistical correlations between averaged behavior variables and cytokine levels were performed in each experiment scenario by Spearman method. Statistical significance was considered at p < 0.05 and *p < 0.05, **p < 0.01, ***p < 0.001, and ****p < 0.0001 convention was used in the presented figures. All statistical analyses were performed with GraphPad Prism 7.

#### Nanostring Analysis: ROSALIND^®^ Nanostring Gene Expression Method and Analysis.

Data was analyzed by ROSALIND^®^ (https://rosalind.bio/), with a HyperScale architecture developed by ROSALIND, Inc. (San Diego, CA). Read Distribution percentages, violin plots, identity heatmaps, and sample MDS plots were generated as part of the QC step. Normalization, fold changes and p-values were calculated using criteria provided by Nanostring. ROSALIND^®^ follows the nCounter^®^ Advanced Analysis protocol of dividing counts within a lane by the geometric mean of the normalizer probes from the same lane. Housekeeping probes to be used for normalization are selected based on the geNorm algorithm as implemented in the NormqPCR R library^39^. Abundance of various cell populations is calculated on ROSALIND using the Nanostring Cell Type Profiling Module. ROSALIND performs a filtering of Cell Type Profiling results to include results that have scores with a p-Value greater than or equal to 0.05.

Differential gene expression analysis was carried out using Student’s t-test on log2-transformed and normalized gene expression. P-value adjustment was performed using the Benjamini-Hochberg method of estimating false discovery rates (FDR). Differential genes between groups were visualized using volcano plot with R package EnhancedVolcano. Heatmap of AHR genes was plotted using R package ComplexHeatmap^40^. R software (version 4.2.2 and higher) was used for gene expression analysis.

## Results

### AHR expression is increased in microglia (MG) from post-mortem brain samples of stroke patients.

**Figure 1:**
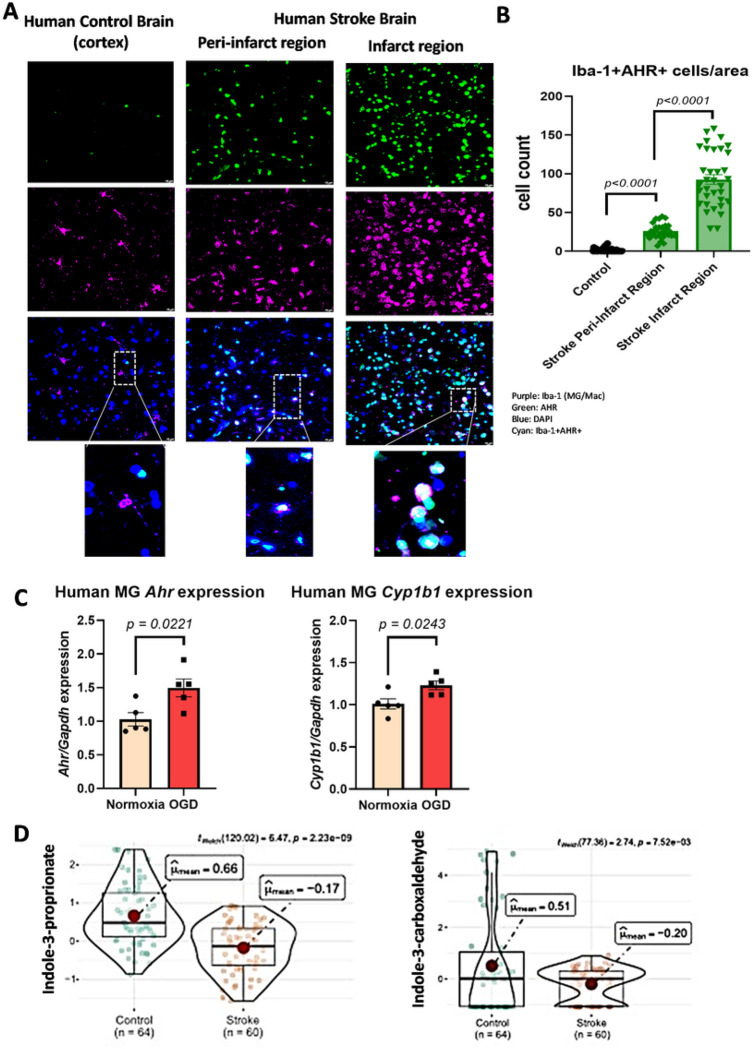
AHR expression is increased in microglia (MG) and other Iba-1+ immune cells in post-mortem brain samples of stroke patients and MG AHR is activated after OGD/R using human cell lines. (**A**) Representative images of double immunofluorescent labeling for AHR (green) and Iba-1 (pink) in brain sections show AHR expression in Iba-1+ cells. (**B**) Quantification of AHR+ MG per unit area in the infarct and peri-infarct regions post-stroke (n=29) compared to cortical regions from controls (n=10). Each dot represents a patient. (**C**) RT-PCR demonstrating increased *Ahr* and *Cyp1ba* expression levels after OGD/R performed on human MG cell line (HMC3). (**D**) Plasma concentrations of indole-3-carboxaldehyde and indole-3-propionate are significantly decreased 24 hours after stroke in humans.

Immunohistochemical analysis of brain samples from stroke patients showed significant increase in expression of AHR in Iba-1 + cells (MG and other myeloid cells) after stroke, compared to non-stroke age-matched controls (Fig. 1A–B). Human MG cell lines (HMC3) that underwent *ex vivo* OGD/R for 2 hours followed by reperfusion-like conditions for 4 hours showed significant increase in expression of Ahr and its transcriptional activity, assessed by expression of *Cyp1ba* (Fig. 1C). Plasma levels of microbiota-derived indole-based ligands of AHR were significantly reduced in samples from human stroke patients compared to controls (Fig. 1D). Plasma concentration of indole-based AHR ligands and stroke severity, as assessed by the National Institute of Health (NIH) Stroke Scale^41^, showed no significant correlation (**Supp Fig S1**).

### The supply of microbiota-dependent indole-based AHR ligands was disrupted after stroke.

**Figure 2:**
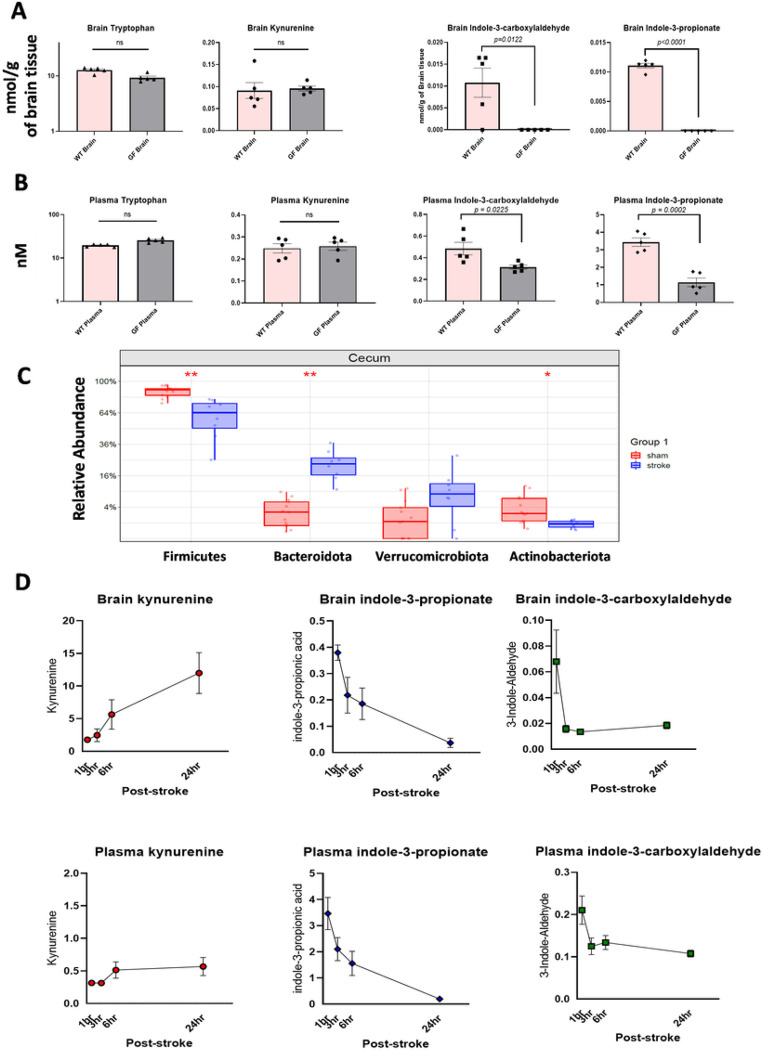
The supply of microbiota-derived AHR ligands is disrupted after stroke in aged WT mice. (**A**) Metabolomics analysis indicates specific indole-based AHR ligands that depend on the presence of a microbiota. Indole-3-carboxaldehyde and indole-3-propionate were undetectable in germ free (GF) brains compared to WT controls, while tryptophan and kynurenine levels did not differ. (**B**) Metabolomics analysis on plasma samples from WT and GF mice. Host-derived metabolites (tryptophan and kynurenine did not differ between WT and GF plasma samples, however, indole-3-carboxaldehyde and indole-3-propionate were significantly reduced in GF plasma. (**C**) 16S data from cecal content of aged mice shows decreased relative abundance in bacterial phyla that include major AHR ligand producers, specifically *Firmicutes* and *Actinobacteria*,post-stroke. (**D**) Metabolomics analysis of aged WT brain and plasma samples at multiple timepoints post-stroke. Kynurenine concentrations increased in the brain and plasma while concentrations of indole-3-carboxaldehyde and indole-3-propionate were significantly decreased in both brain and plasma.

To determine whether brain and plasma concentrations of specific indole-based AHR ligands depend on the presence of commensal microbiota in mice, we performed metabolomics analysis on age-matched brain and plasma samples from WT and GF mice. At least two known indole-based AHR ligands (indole-3-propionic acid (IPA) and indole-3-aldehyde aka indole-3-carboxyaldehyde (IAld)) were undetectable in the GF brain and significantly reduced in the GF plasma when compared to WT samples (Fig. 2A–B). In contrast, the levels of Kyn and Trp were not different between GF and WT brains (Fig. 2A–B), indicating those are host-and diet-derived, respectively. Using 16S rRNAseq, we found that major bacterial populations involved in regulation of AHR ligands and Trp metabolism (e.g., *Bifidobacteriales* and *Lactobacillales*) are significantly reduced with both aging and after experimental stroke in aged WT mice (Fig. 2C, **Supp Fig S2**).

Given the well-known ligand-specificity of AHR^14^, we evaluated the changes in host-derived and microbiota-derived ligands of AHR in the brain after stroke at multiple time points in aged WT mice. We found a significant increase in Kyn levels, which is consistent with previous reports^18^. Importantly, the concentrations of IAld and IPA decrease in both the plasma and the brain after stroke, indicating that stroke induces a loss of the microbiota-derived supply of indole-based ligands of AHR (Fig. 2D).

### Host-derived and microbiota-derived ligands of AHR showed opposing effects on MG survival after OGD/R.

**Figure 3:**
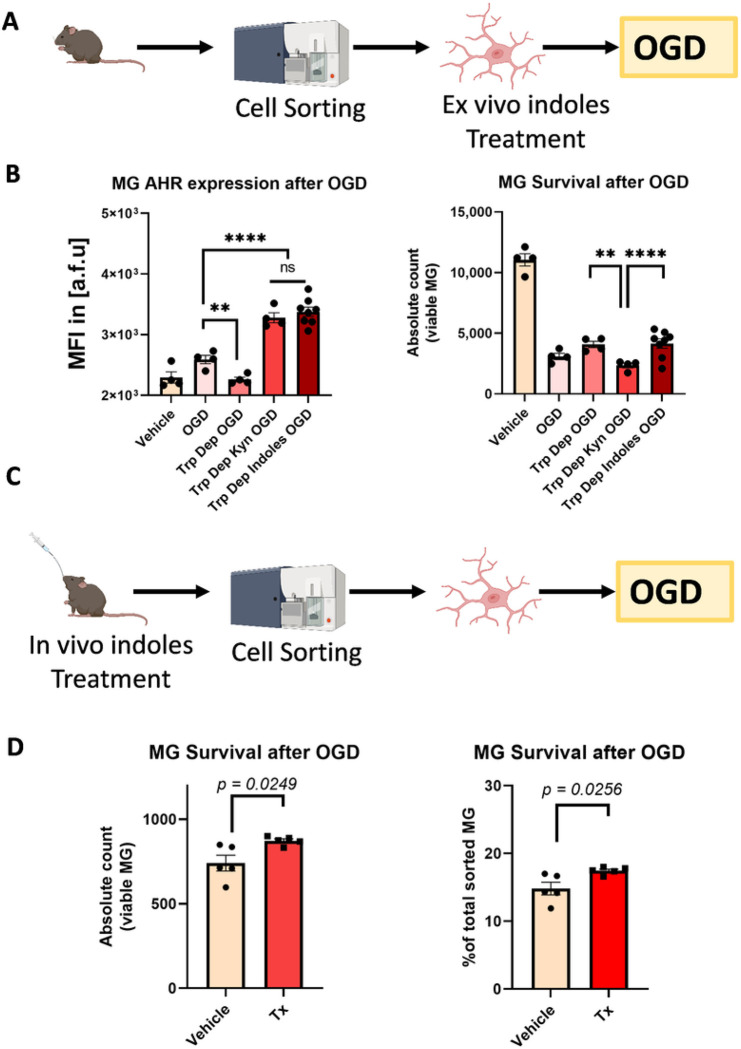
Host-derived and microbiota-derived ligands of AHR have opposing effects on MG survival after oxygen-glucose deprivation and reperfusion (OGD/R). (**A**) Schematic showing sorted MG cells from naive aged WT mice that were treated with indole-based AHR ligands (indole-3-carboxaldehyde and indole-3-propionate) *ex vivo*. The cells then received OGD/R to model stroke conditions. (**B**) MG AHR expression and MG survival post-OGD/R differ based on which AHR ligands are present. AHR expression was increased after OGD/R and depleting tryptophan (Trp) from OGD/R media reversed this affect. The addition of either Kynurenine (Kyn) or indole-based AHR ligands (indole-3-carboxaldehyde and indole-3-propionate) led to an increase in AHR expression post-OGD/R. MG survival after OGD/R was significantly lower when Kyn was present compared with Trp-depleted controls. Post-OGD/R MG survival increased when indole-based ligands were added. (**C**) Schematic showing treatment of mice with indoles(indole-3-carboxaldehyde and indole-3-propionate) *in vivo* via oral gavage. MG were then sorted from indole-treated mice and received OGD/R. (**D**) MG survival was increased post-OGD/R in the group treated with indoles when compared to the vehicle group. The figures were created in BioRender.

We examined whether indole-based and Kyn-based ligands of AHR have different effects on MG survival after OGD/R (Fig. 3A). 2-hour *ex vivo* OGD/R of sorted MG from naïve aged WT mice showed activation of AHR after OGD/R (Fig. 3B). Depleting Trp from the media prior to OGD/R prevented AHR activation after OGD/R, indicating the Trp conversion to Kyn as the predominant mode of AHR activation in the absence of indole-based ligands (Fig. 3B). Adding either Kyn or a cocktail of IAld and IPA to the Trp-depleted media led to a significant increase in AHR expression levels after OGD/R, consistent with the partial agonistic activity of both classes of ligands. Importantly, cell survival of sorted MG after OGD/R was significantly lower when Kyn was added, compared to Trp-depleted controls. In contrast, adding the IAld and IPA cocktail had no significant effect on MG survival after OGD/R (Fig. 3B).

To provide more translational value to the *ex vivo* findings, non-stroke aged WT mice were administered a cocktail of IPA and IAld *in vivo* via oral gavage, then MG were sorted 24 hours after treatment, and *ex vivo* OGD/R was performed on the MG sorted from treated mice (Fig. 3C). Cell survival after OGD/R was significantly higher in MG sorted from mice treated with the cocktail of IPA and IAld compared to MG from mice that received vehicle (Fig. 3D). MG AHR expression was increased after OGD/R, and host- derived and microbiota-derived ligands of AHR had differential effects on MG survival after OGD/R. Specifically, Kyn-mediated activation of AHR was detrimental, while indole-mediated activation of AHR did not worsen MG survival after OGD/R. Next, we tested the effects of post-stroke supplementation with microbiota-derived ligands of AHR *in vivo* using GF and aged WT MCAO models of stroke.

### Post-stroke treatment of microbiota-dependent indole-based ligands of AHR regulated MG-mediated neuroinflammation and antigen presentation molecules in GF mice.

To understand the importance of the commensal microbiota-derived ligands of AHR after stroke, we used GF animal models that are devoid of any bacteria, and thus are unable to convert Trp to indole-based molecules. To confirm the treatment effect, we performed metabolomics analysis of the brain and plasma, which showed increased concentrations of the administered indole-based molecules (**Supp Fig S3**). This confirmed that peripheral supplementation of indole-based ligands is sufficient to increase brain levels of these metabolites.

**Figure 4:**
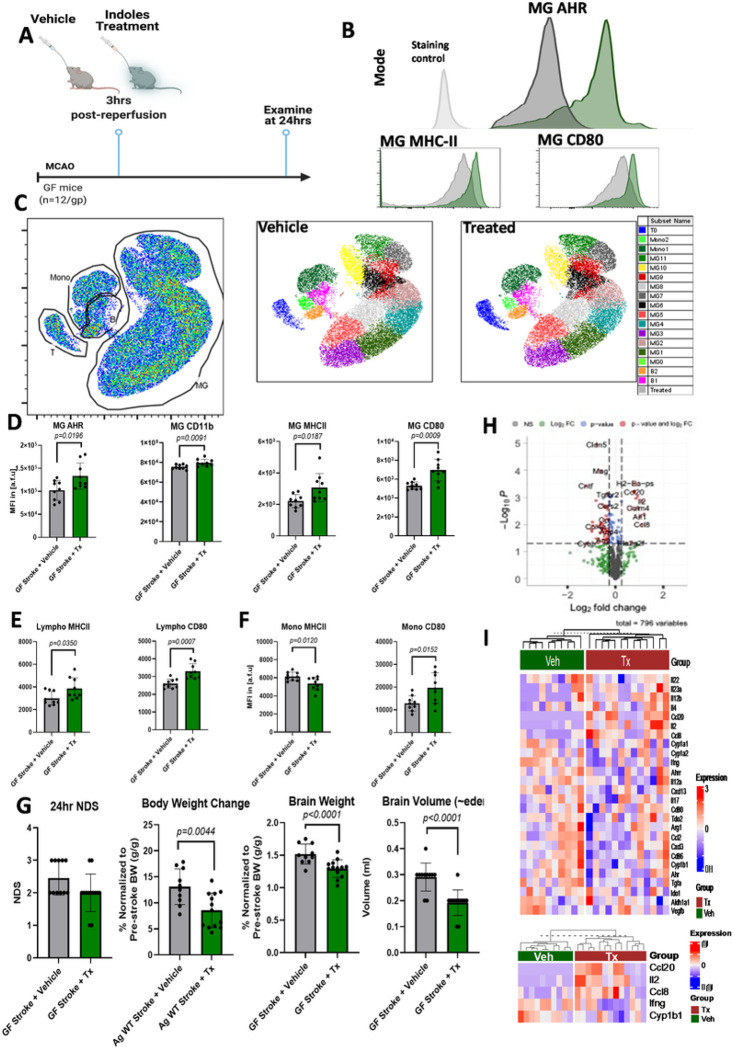
Post-stroke treatment of microbiota-derived indole-based AHR ligands regulates MG-mediated neuroinflammation and surface molecules directly involved in antigen presentation and immune co-stimulation in GF mice. (**A**) Schematics showing timeline of MCAO and indoles treatment in GF mice. (**B**) Increased expression of MG AHR is associated with increased expression of antigen presenting and co-stimulatory molecules MHC-II and CD80. (**C**) Phenographs of non-MG and MG cells of homogenized brains of GF mice post-stroke from vehicle and indoles-treated mice. (**D**) Surface expression of AHR, CD11b, MHC-II, and CD80 by MG are significantly increased at 24 hours after stroke in GF mice treated with indoles 3 hours after stroke compared to GF stroke mice receiving vehicle. (**E**) Surface expression of MHC-II and CD80 by lymphocytes were significantly increased in mice treated with indoles. (**F**) Surface expression of MHC-II is significantly decreased and expression of CD80 is increased by monocytes in mice treated with indoles. (**G**) GF stroke mice treated with indoles had significantly smaller reduction in body weight (normalized to pre-stroke body weight (BW) at 24 hours after stroke, lower brain weight (normalized to body weight at 24 hours post-stroke), and lower brain volume. Neurological deficit scores were not different at 24 hours post-stroke in GF mice. (**H, I**) Volcano plots and heatmap visualization of Nanostring mRNA expression analysis of homogenized brain tissues from the controls and indoles-treated groups of GF stroke mice showing significantly different transcriptional profiles of AHR downstream target genes (*Cyp1b1, Ifng, Ccl8, Il2*, and *Ccl20*) in the indole-treated group. The figures were created in BioRender.

The brains of GF mice subjected to MCAO were immunophenotyped using flow cytometry. Post-stroke treatment with IAld and IPA 3 hours post-reperfusion was associated with increased MG AHR expression (Fig. 4A). This increase in AHR expression was associated with a higher surface expression of CD11b, MHC-II, and CD80 by MG at 24 hours after MCAO compared to the GF MCAO vehicle group (Fig. 4B and 4D). Unsupervised analysis of the flow cytometry data with respect to the seven surface parameters of CD45, CD11b, MHC-II, CD80, P2RY12, and Tmem119 detected several subsets of MG, lymphocytes and infiltrating monocytes visualized as a phonographic comparison of the vehicle and treated GF stroke brains (Fig. 4C). Both heatmap of surface expression levels and fluorescence intensity plots of the cell populations detected by the phonograph algorithm have been included as supplementary materials (**Supp Fig S4 and S5**).

Examination of non-MG immune populations in the brain revealed that lymphocytes had higher expression of MHC-II and CD80 (Fig. 4E), while monocytes exhibited higher expression of CD80 and lower expression of MHC-II in the treatment group compared to the vehicle group (Fig. 4F). Brain weights and volumes were significantly reduced in GF MCAO mice treated with IAld and IPA compared to the vehicle group (Fig. 4G). Nanostring analysis of over 700 genes, including AHR downstream target genes, was performed to assess the transcriptional profile of homogenate brain tissue, which showed significant effects by treatment status upon the visualization by the volcano plot and heatmap of the data (Fig. 4H and I).

### Post-stroke treatment with microbiota-dependent indole-based AHR ligands reduced infarct size and neurological deficits in aged WT mice.

**Figure 5:**
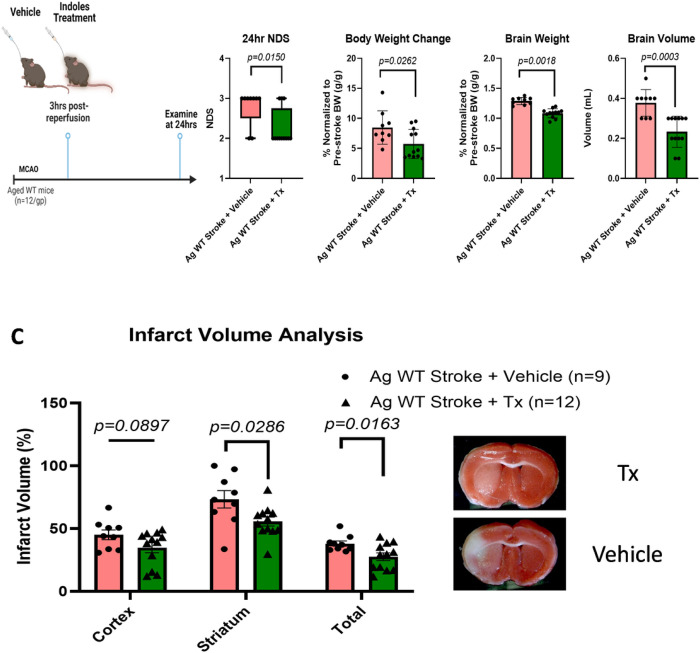
Post-stroke treatment with microbiota-derived indole-based AHR ligands (indole-3-carboxaldehyde and indole-3-propionate) reduces neurological deficits and infarct size in aged WT mice 24 hours after stroke. (**A**) Schematic showing the timeline of MCAO and indoles treatment in aged WT mice. (**B**) Aged WT mice treated with indoles had a significant decrease in neurological deficit scores (NDS), smaller reduction in body weight (normalized to pre-stroke body weight (BW)) at 24 hours after stroke, lower brain weight (normalized to body weight at 24 hours post-stroke), and lower brain volume. (**C**) Quantification of brain infarct volumes as analyzed by TTC staining in controls (n=9) vs treated (n=12) mice. There is a significant decrease in total, cortical, and striatal infarct size in the stroke mice treated with indoles compared to the vehicle group. The figures were created in BioRender.

Lastly, we examined the effect of post-stroke treatment with indole-based AHR ligands in aged WT mice (Fig. 5A). Neurological deficit scores (NDS) in aged WT MCAO mice treated with IAld and IPA 3 hours post-reperfusion were significantly lower than the aged WT MCAO vehicle group (Fig. 5B). Aged WT MCAO mice treated with IAld and IPA, compared to the vehicle group, had less body weight (normalized to pre-stroke body weight (BW) reduction at 24 hours after stroke, lower brain weight (normalized to body weight at 24 hours post-stroke), and lower brain volume (Fig. 5B). Infarct volume analysis of aged WT brains showed that the mice treated with the indole-based ligands of AHR had significantly smaller total infarct volumes than the vehicle group (Fig. 5C).

Taken together, these novel results strongly support that restoring a balanced pool of host-derived kynurenine-based and microbiota-derived indole-based ligands of AHR via post-stroke administration of indole-based ligands of AHR can improve stroke outcomes in mice through the regulation of MG-mediated neuroinflammation and surface molecules directly involved in antigen presentation and immune co-stimulation.

## Discussion

In the present study, we utilized human brain and plasma samples and GF and aged WT mouse models of ischemic stroke. We investigated the effects of post-stroke treatment with microbiota-derived indole-based AHR ligands. We focused our investigation of these effects to MG-mediated neuroinflammation and surface molecules involved in antigen presentation and immune co-stimulation. Our results showed that MG AHR expression is increased after stroke in humans. Post-stroke treatment with indole-based AHR ligands beneficially regulated MG-mediated antigen processing and co-stimulatory immune functions. Treatment with indole-based molecules to restore a balanced pool of host-derived and microbiota-derived AHR ligands reduced infarct size and neurological deficits in aged WT mice.

### AHR is a ligand-specific mediator of the host-microbiota interactions.

AHR is a highly conserved, ligand-activated transcription factor that regulates both immune differentiation and neuroinflammation^9^. AHR integrates environmental, dietary, metabolic, and microbial cues to regulate essential immune homeostasis and inflammatory conditions^9,42,43^. AHR binds structurally diverse exogenous and endogenous compounds, including environmental toxins such as dioxins, phytochemicals such as flavonoids, host-derived Trp-based molecules such as kynurenines, and microbiota-derived indole derivatives^44^. Most AHR ligands are low molecular weight, lipophilic polycyclic planar compounds^14^, which can easily pass through critical physiological barriers such as the intestinal epithelial barrier and blood-brain barrier (BBB)^14, 45–48^. A major challenge in investigation of AHR immunobiology is that the role of AHR is highly disease-, tissue-, cell- and ligand-specific^9^.

### Endogenous sources of AHR ligands:

#### Host-derived Trp-based AHR ligands:

Circulating Trp is predominantly albumin-bound, which prevents its BBB crossing^49,50^. Free Trp is transported from the blood across BBB by a competitive, non-specific transporter of large neutral L-amino acids^51,52^. Trp is converted to Kyn by the host enzymes TDO/IDO under the influence of IL-1β and IL-6^53^. Levels of TDO/IDO increase after stroke and pharmacological inhibition of their enzymatic activity reduces AHR activation after stroke^18^. Importantly, Kyn pathway metabolites are detected in GF mice^54,55^, also validated in our study, indicating those are host-derived even in the complete absence of gut microbiota.

### Microbiota-derived Trp-based AHR ligands

Another major class of AHR ligands, primarily regulated by gut microbiota enzymes (e.g., bacterial tryptophanase), are the Trp-derived indole-based AHR ligands^45,56,57^. Trp is the only amino acid that contains an indole structure. Indole production is dependent on microbial catabolic activity of dietary Trp^46,58^. Indoles are aromatic heterocyclic molecules with planar structure formed by fusion of a benzene and a nitrogenous pyrrole ring, which makes them structurally distinct from Trp-derived Kyn-based ligands of AHR. Indole-based metabolites can be converted into various AHR ligands via hepatic uptake and metabolism^9^. Most indole-based AHR ligands are partial agonists, meaning they can serve as competitive antagonists in the presence of other partial agonists, outcompeting the detrimental effects of Kyn and other host-derived AHR ligands^59^, which is an important premise of the present study.

### Limitations of knock-out mouse models for testing our hypothesis

Generating a global or cell-specific (e.g., *Ahr* deletion in MG) will not allow testing the hypothesis of this study as various AHR ligands share the same set of ligand binding domains. In other words, any knock-out model that eliminates activation of AHR by both Kyn-based and indole-based ligands would not allow testing whether indole-based ligands can reduce detrimental effects of kyn-based ligands.

### Indole-based ligands have a higher affinity and potency for human AHR compared to mouse AHR.

Importantly, many endogenous Trp-derived AHR modulators (not exogenous toxins), particularly indole-based molecules, have greater potency (activation potential) for human AHR compared to rodent AHR^14,15,58^. The increased affinity of these endogenous AHR ligands implies an evolutionary adaptation in human host cells, allowing them to be influenced by a diverse microbiota, which in turn mediates physiological responses, including inflammation. For example, the uremic toxin indoxyl-3-sulfate (I3S) shows a 500-fold greater potency in activating human compared to murine AHR hepatoma cell lines^60^, and therapeutic approaches including AHR antagonism or inhibition of I3S synthesis have been proposed as treatments for chronic kidney disease^61,62^. Indole itself is a potent agonist of human AHR while only a weak agonist of mouse AHR^15^. Accumulating data indicate that human AHR has a higher affinity for its indole-based ligands than mouse AHR does^13^, and our *ex vivo* experimental data confirmed activation of MG AHR after OGD/R. This suggests that there may be even greater therapeutic benefits from microbiota-based modulation of AHR in humans than in mice.

### MG AHR is a major regulator of neuroinflammation.

Microbiota-regulated Trp-derived ligands activate MG AHR to suppress activation of the NF-κB pathway. MG AHR activates the TGF-α promoter which interferes with NF-kB-driven expression of VEGF-β^63^. MG TGF-α and VEGF-β suppress and induce astrocyte-mediated inflammation, respectively^9^. Deletion of MG AHR exacerbates neuroinflammation and increases the recruitment of inflammatory monocytes to the brain^63^. MG-specific *Ahr* deletion is detrimental in chronic neuroinflammation models such as experimental autoimmune encephalomyelitis^63^, suggesting that the MG AHR is required for anti-inflammatory effects of indole-based AHR activation. Our results demonstrated that host-derived Kyn-based and microbiota-derived indole-based molecules activated MG AHR but have different effects on MG survival after OGD/R. Additionally, our results showed that MG antigen presentation and co-stimulation signaling molecules are significantly increased when GF mice were treated with the cocktail of IPA and IAld after stroke, indicating that MG-mediated neuroinflammation can be modulated by post-stroke administration of indoles.

### Kyn-mediated activation of MG AHR is detrimental after stroke.

Kyn is increased in the brain within hours after stroke and promotes deleterious effects in cerebral ischemia by activating AHR^18^. Pharmacological AHR inhibitors or deletion of the *Ahr* gene in global knock-out models decreases ischemic damage and improves neurological deficit scores in young male mice^12,18^. AHR activity is increased in the brain after ischemia in mice^18^. Post-stroke administration of two different pharmacological antagonists of AHR reduced infarct volume at 48hrs. Additionally, administration of Kyn significantly increased infarct volume and this effect was not observed with co-administration of pharmacological AHR antagonist or in *Ahr*−/− mice^18^. These previously reported results clearly confirmed the detrimental effects of the Kyn-AHR pathway in acute stroke and, more importantly, the post-stroke responsiveness of the brain AHR pathway to peripherally-sourced molecules (Kyn or pharmacological inhibitors^18^). Our results showed that post-stroke changes in the gut microbiota led to a loss of microbially-derived indole-based ligands of AHR. These compounds serve a similar role as do pharmacological inhibitors of AHR due to their partial agonistic properties. Thus, we hypothesized that increasing the concentration of microbiota-dependent indoles in the brain would ameliorate the deleterious effects of Kyn-based activation of AHR by providing a competitive beneficial ligand. As mice with global deletion of *Ahr* (*Ahr*−/− KO) or mice with MG-specific deletion of *Ahr* eliminates the cellular receptor for both indole-based and kyn-based ligands of AHR, these models are not appropriate to test our overall hypothesis.

### MG AHR expression is increased after human stroke and microbiota-dependent supply of AHR ligands is disrupted.

We began by demonstrating the significant expression (known to be an indirect measure of activation) of AHR in MG and significant reduction of plasma levels of microbiota-derived indole-based ligands of AHR after human stroke. In contrast to decreases in the indole-based ligands of AHR after stroke, increased brain and plasma concentration of host-derived Kyn-based AHR ligands has been reported^18^. However, large datasets reporting alterations of both kyn-based and indole-based ligands AHR after human stroke are not currently available. This clinical data gap is of high translational relevance in stroke immunology as multiple studies have reported that indole-based molecules have a significantly higher affinity, at physiological concentrations, for the human AHR compared to mouse AHR^15,64^.

### Brain concentration of indole-based AHR ligands is dependent on a functional microbiota.

It has been reported that IPA is not detectible in plasma from GF mice^46^, but similar analysis of brain tissue had been performed prior to this study. We identified two indole-based AHR ligands (IPA and IAld) that were undetectable in the GF brain. Additionally, we showed that known AHR ligand producers are decreased in the gut microbiota with aging and after stroke in aged WT mice, consistent with the existing microbiome literature. For example, relative abundance of *Bifidobacterium* is significantly decreased in fecal samples collected 24 hours after acute ischemic stroke patients^65^. *Bifidobacterium* abundance and diversity decreases with age^66–68^. *Bifidobacterium* and *Lactobacillus* abundance levels decrease after experimental stroke in rats^69^.

These findings allowed us to investigate the effect of post-stroke treatment with indole-based AHR ligands in both GF and aged WT mice after stroke. We evaluated multiple time points after stroke and observed a step-wise reduction of IPA and IAld in both the plasma and the brain, suggesting a loss of the bottom-up supply (meaning produced by the gut microbiota and detected in the plasma and brain) of these microbiota-dependent AHR ligands. This reduction of indole-based AHR ligands is likely due to the stroke-induced dysbiosis of the gut microbiota, which has been extensively reported by our group and others^26,28,32^. Moreover, we verified the previously-reported^18^ increase in the brain Kyn levels as a representative molecule of the host-derived AHR ligands produced along the Kyn pathway^12^.

### MG survival after ischemia is worsened with Kyn but not with indole-based AHR ligands.

Importantly, our *ex vivo* results indicated a ligand-specific effect of Kyn and indole-based AHR ligands on MG survival after OGD/R, suggesting a dichotomy between the host-derived Kyn and microbiota-derived indoles in the context of cellular stress such as OGD/R. We then examined this beneficial effect of indole-based AHR ligands on MG survival via *in vivo* administration of indole-based AHR ligands followed by *ex vivo* OGD/R on the sorted MG. Our *ex vivo* data clearly demonstrated the ligand-specificity of AHR, which has been extensively reported in other disease conditions^14,70^. Our results showed the effects of adding Kyn to a Trp-depleted media was detrimental, while adding IPA and IAld cocktail to a Trp-depleted media was not detrimental. We hypothesize that indole-based AHR ligands exert their beneficial effects primarily as partial agonists of AHR, outcompeting the detrimental effects of Kyn and other host-derived AHR ligands; consequently, in the absence of Trp and Kyn, they may not exhibit an independent beneficial effect. The pool of endogenous ligands of AHR can be viewed as a balance of host-derived and microbiota-derived molecules, the disturbance of which can be a source of detrimental immune response after stroke^71,72^.

### Post-stroke treatment of microbiota-derived indole-based ligands of AHR is beneficial.

As a final proof of principle, we concluded with a cohort of GF and aged WT stroke mice. These experiments (Figs. 4 and 5) were conducted in parallel to minimize experimental variability. Our results showed that post-stroke treatment of GF mice with a cocktail of IPA and IAld significantly increases expression of MG AHR, MHC-II, and CD80. The surface expression of MHC-II and CD80, regulated by AHR transcriptional activity^9,15^, are direct markers of antigen processing and the co-stimulatory function of MG and other immune cells. Indeed, our results indicated a concerted regulation of surface phenotype by MG, brain lymphocytes, and infiltrating monocytes 24 hours after stroke in GF stroke mice treated with IPA and IAld compared to GF stroke mice treated with vehicle. Lastly, using aged WT mice as a translational preclinical model of stroke, we showed that infarct volume and neurological deficits were significantly reduced by post-stroke treatment with IPA and IAld.

In conclusion, both host-derived and microbiota-derived ligands play a role after stroke by activating AHR in MG and other immune cells. Due to the highly ligand-specific nature of AHR immunobiology and the partial agonistic activity of these endogenous ligands, a cooperative/competitive type response can be expected from AHR in presence of a balanced pool of ligands. As shown here, this balance of AHR ligands is disturbed by the immediate post-stroke decrease in the microbiota-dependent supply of indoles and increase in host-derived Kyn, which have detrimental effects after stroke. Our results do not exclude indirect beneficial effects of indole-derived ligands of AHR on post-stroke neuroinflammation. Future experiments are needed to investigate the role of immune cross-talk between MG and astrocytes, MG and lymphocytes, and MG and endothelial cells under the influence of microbiota-derived indole-based ligands of AHR. Additional studies for long-term stroke outcomes and behavioral differences in stroke mice of both sexes treated with indole-based AHR ligands are warranted. Our results demonstrate that restoring a balanced pool of host-derived and microbiota-derived molecules via post-stroke treatment with indole-based AHR ligands to compete with Kyn-based activation of AHR provides a net beneficial effect and improved post-stroke outcomes.
